# Primary Hyperparathyroidism in Older Adults: A Narrative Review of the Most Recent Literature on Epidemiology, Diagnosis and Management

**DOI:** 10.3390/jcm12196321

**Published:** 2023-09-30

**Authors:** Youssef Rizk, Nour Saad, Wassim Arnaout, Moussa A. Chalah, Stephanie Farah

**Affiliations:** 1 Department of Internal Medicine, Division of Family Medicine, LAU Medical Center-Rizk Hospital, Gilbert and Rose Marie Chagoury School of Medicine, Lebanese American University, Beirut P.O. Box 13-5053, Lebanon; youssef.rizk90@gmail.com (Y.R.); saadnour06@gmail.com (N.S.); wassimarnaout@gmail.com (W.A.); 2 Gilbert and Rose-Marie Chagoury School of Medicine, Lebanese American University, Byblos P.O. Box 13-5053, Lebanon; 3 Institut de la Colonne Vertébrale et des Neurosciences (ICVNS), 75116 Paris, France; 4 Endocrinology, Diabetes and Metabolism, Private Practice, Haddade Street, Batroun P.O. Box 1400, Lebanon; 5 Division of Research, LAU Medical Center-Rizk Hospital, Gilbert and Rose-Marie Chagoury School of Medicine, Lebanese American University, Byblos P.O. Box 13-5053, Lebanon

**Keywords:** primary hyperparathyroidism, elderly, prevalence, clinical manifestations, medical management, surgery, complications

## Abstract

Background: Primary hyperparathyroidism (PHPT) is a common endocrine disorder among older adults. The aim of this review is to shed light on PHPT, particularly in this age group, in terms of prevalence, clinical manifestations, medical and surgical management, and post-operative complications. Methods: Eligible studies were those considering PHPT exclusively in the older population (main databases: PubMed, Medline, Google Scholar and the University Online database). Articles published in the last 10 years (2013–2023) were considered. Eligibility criteria followed the SPIDER (sample, phenomenon of interest, design, evaluation, research type) tool. The methodological quality of the studies was assessed using the Joanna Briggs Institute critical appraisal tool. A total of 29 studies (mainly observational) matched the inclusion criteria. Results: The prevalence of PHPT is approximately 1 per 100 in the elderly, and it is more common in females. The clinical presentation varies by age and can include osteoporosis, fractures, and neuropsychiatric symptoms. Conservative management can be an option whenever surgery is not indicated or feasible. However, parathyroidectomy (PTX) remains a safe and effective modality in aging populations with improvement to symptoms, bone mineral density, fracture risk, frailty, quality of life, and metabolic derangements. Complication rates are similar in elderly people compared to younger ones, except for mildly longer length of hospital stay and reoperation for those with higher frailty. Conclusion: PHPT is a common yet overlooked and underdiagnosed condition among the older population. The safety and efficacy of PTX in the older population on different levels is now well demonstrated in the literature.

## 1. Introduction

Primary hyperparathyroidism (PHPT) is a common endocrine disorder among older adults, primarily affecting patients 65 years and older [1]. The prevalence of PHPT has been increasing during the past three decades [1], with recent evidence showing that about 1.5% of patients over the age of 70 have PHPT [2]. The incidence of PHPT increases with age, ranging from 12–24 per 100,000 in patients aged <50 years, to 95–196 per 100,000 in patients aged 70–79 [1].

PHPT is characterized by hypercalcemia and elevated serum levels of parathyroid hormone (PTH) caused by an excessive production of PTH from one or more of the parathyroid glands [3,4]. PHPT is thought to be the most common cause of hypercalcemia [1]. The mechanism by which increased levels of PTHs lead to hypercalcemia occurs via increased renal tubular calcium reabsorption, rapid mobilization of calcium and phosphate from bone (bone resorption), and increased renal synthesis of 1,25(OH)2D, which in turn increases intestinal calcium and phosphate absorption [5]. The most common pathological finding in patients with PHPT (~80% of patients) is a solitary parathyroid adenoma, with higher rates of multiglandular disease in older adults [6,7]. The only curative treatment is parathyroidectomy.

The manifestation of PHPT is classically described as one targeting the skeletal and renal systems, with patients commonly presenting with reduced bone mineral density (BMD) or fragility fractures, as well as kidney stones, nephrocalcinosis, or renal insufficiency [8]. However, the introduction of routine serum calcium measurements in regular metabolic panels has led to a change in how PHPT manifests, previously as a symptomatic disease and now as an asymptomatic presentation; approximately 70–80% of patients now present as asymptomatic [9,10]. While most patients are asymptomatic and have mild hypercalcemia, patients may have evidence of subclinical skeletal sequelae, such as osteoporosis and vertebral fractures, as well as renal sequelae, such as hypercalciuria and nephrolithiasis, both of which are commonly asymptomatic [11]. PHPT has also been associated with hypertension, angina, myocardial infarction, and arrhythmias, which in turn increase the risk of mortality [12,13,14,15]. Neuropsychiatric symptoms have been also reported in the context of PHPT early in the literature [16], and their underlying mechanisms might involve parathyroid hormone, calcium, neurotransmission enzymes (tyrosine hydroxylase, monoamine oxidase), cytokines (interleukin-6), and ion transporters (sodium-potassium adenosine triphosphatase transporter) [17]. Such manifestations include (a) personality/behavioral changes; (b) depressive, manic, anxious, psychotic, catatonic, and obsessive–compulsive symptoms; (c) neurocognitive deficits (namely memory) leading sometimes to dementia diagnosis; and (d) confusion, delirium, or even coma [17,18,19]. Parkinsonism has also been described in the context of PHPT, but whether it is related to PHPT or an incidental finding in its context remains to be elucidated [20]. Such symptoms appear clinically relevant to consider, especially in elderly patients, in whom PHPT could present as neuropsychiatric symptoms rather than a typical endocrine manifestation [21].

Individuals who are 65 years and older often have multiple medical conditions and therefore need multiple medications. PHPT can add complexity to their medical management, especially in the presence of age-related complications (e.g., cardiovascular disease, osteoporosis, renal impairment).

As our population continues to age and as average life expectancy increases, the proportion of elderly patients with PHPT will also grow. Though the standard of care to manage PHPT is surgery to achieve cure, the role of surgery in the elderly is less clear. While evidence supporting treatment modalities that result in better outcomes has been emerging, there is a scarcity of guidelines focused on treating hyperparathyroidism specifically in the older population. On that premise, we offer a contemporary comprehensive review focused on the epidemiology, diagnosis, treatment options, and post-operative complications of primary hyperparathyroidism in the older population.

## 2. Materials and Methods

Studies eligible for this review were those targeting PHPT exclusively in the older population (65 years and above) in terms of prevalence/epidemiology, clinical presentation (signs and symptoms), management (whether surgical or medical), and complications. Excluded studies were those not fitting inclusion criteria, studies targeting people younger than 65 years old, or studies based on basic science/animal models. Studies published from 2013 to 2023, in English, and without any geographical restrictions were included. Databases searched were mainly PubMed, Medline, Google Scholar, and the Lebanese American University (LAU) online database. Eligibility criteria followed the SPIDER (sample, phenomenon of interest, design, evaluation, research type) guidelines [22]. The study population was the older population (65 years and above), and the phenomenon of interest was PHPT. The following terms and keywords were used: (“primary hyperparathyroidism“ OR “hypercalcemia” AND (“older people” OR “elderly” OR “aged people” OR “65 years old and above”) AND (“epidemiology” OR “prevalence”) AND (“clinical presentation” OR “signs” OR “symptoms” OR “clinical picture”), AND “management” OR “surgery” OR “parathyroidectomy” OR “medications” OR “medical”) AND (“comorbidities” OR “consequences” OR “complications”). The Institutional Review Board (IRB) was not needed since no patients were recruited and there was no imposed risk. 

Two reviewers (S.F. and Y.R.) performed the literature review independently. Each study was subsequently identified for inclusion after mutual agreement. In case an article did not clearly meet the inclusion criteria based on its title or abstract, or in case of disagreement, the full text was retrieved and reviewed for a definite decision. A study was included when both reviewers agreed on it. No review protocol was published. Data were checked for clarity and were presented in a tabulated form to compare sample size, study design, and outcomes. No assumptions or simplifications were made. Both observational (case report, case series, cohort, case control, and cross-sectional) and interventional studies (Randomized or non-randomized clinical trials) were included. 

The search yielded 225 record hits. We excluded 180 records during initial screening. They were either duplicates, irrelevant to the subject, exclusively targeting patients below 65 years old, or beyond the scope of our review. A total of 29 articles met the inclusion criteria (Table 1). The studies break down as follows: 5 studies emphasized the epidemiology [7,23,24,25,26], 8 studies were available with regard to the clinical manifestations [25,27,28,29,30,31,32,33], 25 studies addressed the treatments [7,15,23,24,25,27,28,29,30,31,32,34,35,36,37,38,39,40,41,42,43,44,45,46,47], and 7 considered the post-operative complications [7,25,26,37,44,48,49]. The Joanna Briggs Institute (JBI) critical appraisal tool was used to assess the methodological quality of the included studies and the data are presented in Appendix A (Table A1, Table A2, Table A3 and Table A4).

## 3. Results

### 3.1. Epidemiology

Primary hyperparathyroidism is the most common cause of hypercalcemia [23] with a prevalence of approximately 1 per 100 in the elderly [24]. It mostly occurs in elderly females [25,26]. Multiglandular disease is more frequent in older patients compared to younger ones, with a rate of 20–30% in the former group [7].

### 3.2. Clinical Manifestations

The clinical presentation of PHPT varies by age. Persons above 65 years old are most likely asymptomatic or present with osteoporosis, mainly at the level of the forearm and cortical bones, [25,27]. Other somatic complaints, including constipation, nausea, arthralgia, and acute hypercalcemia, were also described [25]. On the other hand, nephrolithiasis and osteitis fibrosa cystica were mainly observed in younger patients. [25].

Patients can also exhibit cognitive, affective, and behavioral manifestations as well as movement disorders [25,28,29,30,31,32] with even mild hypercalcemia [33]. Neuropsychiatric symptoms include memory loss, disorders of consciousness, sleep disturbance, fatigue, anxiety, and depression, as well as manic, psychotic, and obsessive–compulsive symptoms, among others.

### 3.3. Conservative Management

Older patients with mild asymptomatic disease can be safely managed with a conservative approach and a thorough follow-up with scheduled visits every 6 months and yearly bone densitometry (BMD), especially when the surgical risk outweigh its benefits [24,27,34]. In one case report with hypercalcemia and mixed Alzheimer’s and vascular dementia diagnosis, a cinacalcet trial resulted in a significant improvement in cognitive deficits (Mini Mental Status Exam rising from 9 to 21/30) which was accompanied by a gain in autonomy [29].

It is important to note that serum and urinary calcium significantly decreased during follow-up in patients who did not undergo surgery. A retrospective analysis of 141 patients treated conservatively showed that serum and urinary calcium levels had significantly decreased, and vitamin D level had increased at last visit (10.4 ± 0.5 mg/dL, 161 ± 70 mg/24 h, 69 ± 17 nmol/L, *p* < 0.010 respectively) compared with levels at diagnosis (10.6 ± 0.2 mg/dL, 223 ± 95 mg/24 h, 53 ± 15 nmol/L, respectively, *p* = 0.001) [24].

Interestingly, in patients treated with disothiazide (used in cases of hypercalciuria and nephrocalcinosis), serum calcium level decreased (10.5 ± 0.5–10.3 ± 0.4 mg/dL, *p* = 0.050), PTH level did not significantly change (x1.6 ± 0.7 to x1.7 ± 0.8, *p* = 0.400), and the rates of osteoporosis, fractures, and death were 17/23 (74.0%), 11/23 (48.0%) and 2/23 (9.0%), respectively [24].

### 3.4. Surgical Management

The likelihood of parathyroidectomy decreased with increasing age. Surgery was concerning due to multiple comorbidities, patient’s age, presence of mild or asymptomatic disease, stable hypercalcemia [23], and severe frailty [35]. Furthermore, elderly patients, particularly those who are Black, Hispanic, or Asian, have lower rates of undergoing parathyroidectomy compared to elderly White patients [36]. However, on the other hand, opposing data showed that there is a significant increase in the number of patients undergoing parathyroidectomy over the past two decades [37]. Neck cervicotomy and parathyroidectomy were recommended as the preferred safe treatments for elderly patients with PHPT, even for minimal hypercalcemia [15]. Parathyroidectomy was found to be curative in those patients [38,39], with excellent surgical outcomes (80/80 (100.0%) cure in patients ≤50 years compared to 78/80 (97.5%) for patients ≥75 years) (*p* = 0.150) [25].

Parathyroidectomy was significantly associated with lower major adverse cardiovascular events (MACE) (HR 0.92 [95%CI 0.90–0.94]), cardiovascular disease-related hospitalization (HR 0.89 [95%CI 0.87–0.91]), and cardiovascular hospitalization-associated mortality (HR 0.76 [95%CI 0.71–0.81]) compared to non-operative patients [40]. However, the absolute risk reductions (ARRs) associated with parathyroidectomy were significantly modest. ARR for MACE was 1.7% (95%CI 1.3–2.1%), ARR for cardiovascular disease-related hospitalization was 2.5% (95%CI 2.1–2.9%), and ARR for cardiovascular hospitalization-associated mortality was 1.4% (95%CI 1.2–1.6%), and the clinical significance was more evident in the long-term follow-up [40].

Comparing parathyroidectomy to nonoperative therapy at 2, 5, and 10 years, there was an adjusted absolute fracture risk reduction of 1.2% (95%CI, 1.0–1.4), 2.8% (95%CI, 2.5–3.1), and 5.1% (95%CI, 4.6–5.5), respectively [41]. A multivariable Cox analysis showed that a surgical approach was shown to reduce the adjusted HR of any clinical fracture by 16% and the adjusted HR of hip fracture by 17% [42,43]. Additionally, there was an increase in bone mineral density (BMD) in 85.6% of elderly patients compared to 79.8% in a younger population after surgery [42]. Significant improvement from pre-operative T-score was noted in most patients at the first post-operative DEXA scan (*p* = 0.012) as well as at the third post-operative DEXA scan (*p* = 0.026) [44].

In terms of symptomatology, younger groups experienced a greater reduction in mood swings, irritability, itchiness, and feeling thirsty, while the older groups had a greater decrease in bone pain, tiredness, weakness, joint pain, difficulties getting off chairs, headaches, and frailty outcomes [7]. Risk Analysis Index scores decreased after parathyroid exploration surgery, which reflects an improvement in frailty score [45].

Interestingly, short-term normalization of hypercalcemia via calcimimetic treatment and subsequent improvement in depression and anxiety symptoms (according to the Hospital Anxiety and Depression Scale) are good predictors of long-term outcomes after 6 months of parathyroidectomy, especially at the level of the cognitive response (according to the Montreal Cognitive Assessment) and therefore aid in an appropriate patient selection for surgery [46]. It is worth noting that improvement in neurological and psychiatric symptoms following parathyroidectomy was also supported by some case reports [28,30,31,32]. Despite the evidence-based positive outcomes, parathyroidectomy is still underused for symptomatic PHPT in older adults [47].

### 3.5. Post-Operative Complications

There was no difference in the rate of post-operative complications between the younger (<75 years) and the elderly group (≥75 years) in terms of transient recurrent laryngeal nerve injury (0.5% vs. 0%; *p* = 0.300) and permanent recurrent laryngeal nerve injury (1.0% vs. 1.0%; *p* = 0.988), nor in the rates of persistent (3.6% vs. 4.5%; *p* = 0.538) or recurrent (2.6% vs. 1.8%; *p* = 0.505) PHPT post-parathyroidectomy [37]. Recent data showed that older patients had post-operative risk of in-hospital complications comparable to younger patients (50–64 years: odds ratio (OR): 0.51 (95%CI, 0.28 to 0.92) *p* = 0.026; 65–74 years: OR: 0.72 (95%CI, 0.39 to 1.33) *p* = 0.295; ≥75 years: OR: 1.03 (95%CI, 0.54 to 1.95) *p* = 0.936, respectively); as well as intensive care unit (ICU) admissions (50–64 years: OR: 0.91 (95%CI, 0.39–2.14) *p* = 0.832; 65–74 years: OR = 1.06 (95%CI 0.43–2.63) *p* = 0.894; ≥75 years OR: 1.48 (95%CI 0.58–3.74) *p* = 0.413) and 30-day risk of readmission to the hospital (50–64 years: OR 1.02 (95%CI 0.45–2.28) *p* = 0.971; 65–74 years: OR 1.20 (95%CI 0.51–2.82) *p* = 0.68; ≥75 years OR 1.94 (95%CI 0.81–4.61) *p* = 0.136) [26].

On the other hand, elderly people tend to experience more post-operative cardiovascular complications [44] as well as delirium, arthralgia, hypotension, and phlebitis [25]. It is important to mention that patients who have higher frailty, reflected in high modified five-item frailty index (mFI-5) scores, had more than 8 times the odds of experiencing complications such as reoperation within 30 days (OR 4.20, 95%CI 1.64–10.74; *p* = 0.003) and prolonged LOS for 5 days (95%CI 4.28–5.25; *p* = 0.001) compared to those with low scores [48].

Finally, minimally invasive parathyroidectomy has a more successful cosmetic result, lower pain, lower LOS, and lower risk of complications compared to conventional parathyroidectomy [7,49]. Table 2 summarizes the main outcomes of each study.

## 4. Discussion

The incidence of PHPT is expected to increase as a result of a booming elderly population [37]. The condition can go undiagnosed in this age group due to the non-specific signs and symptoms of the disease and its unique manifestation compared with the young and middle-aged age groups. Whereas younger and middle-aged patients present with typical symptoms such as renal, bone, and gastrointestinal manifestations, older patients frequently present with symptoms of accentuated aging, including vague neuromuscular symptoms, fatigue, loss of appetite, constipation, decreased intellectual capacity, psychological problems (e.g., emotional instability, obsessive–compulsive disorder, depression, anxiety, and paranoia), bone disease (e.g., demineralization) and musculoskeletal pain [13,50].

The overlapping clinical picture of aging and PHPT can complicate the situation even further, making PHPT and aging difficult to distinguish for clinicians, while having multiplicative effects [51]. Hypertension secondary to hypercalcemia as result of PHPT can mistakenly be attributed to increased age. Neurocognitive symptoms from PHPT can be difficult to differentiate from age-related decline in neurocognitive capacity and/or other chronic diseases, such as dementia [13,50]. Symptoms such as low mood and fatigue tend to be more associated with aging-related psychiatric disorders, although these problems may also be caused by PHPT [2,52]. In many cases, psychiatric symptoms may be the only symptom in PHPT, and thus clinicians should suspect PHPT in patients with psychiatric symptoms and mild hypercalcemia [33]. Moreover, the systemic effects of hypercalcemia, above and beyond the increased risk of osteoporosis and fractures, are of major clinical concern in the geriatric population, particularly among those who are frail [24]. These risks are even more relevant in the presence of concomitant morbidities that are common in this age group, such as falls, preexisting idiopathic osteoporosis, neurological disorders, chronic use of drugs (diuretic and steroids), poor nutrition, vitamin D deficiency, and cognitive impairment [24,43], which might lead to further morbidity, higher rates of fractures, and worse outcomes. Today, the milder forms of hypercalcemia that are being detected by laboratory tests rather than the classical presentation of “bones, groans, and psychiatric overtones”, are mainly driven by the introduction of routine serum screen, offering the additional advantage of early detection of PHPT [52].

Despite the known benefits of early detection and intervention, there is often a delay in diagnosis and referral to surgery [52,53]. Typical reasons for a delay in diagnosis of hyperparathyroidism in older adults are attributed to the treating physician missing or not acknowledging a high calcium level, attributing hypercalcemia to other causes, or not undertaking a workup to rule out hyperparathyroidism [23]. The most common reasons for surgery delay are the tendency of physicians to defer surgery due to increased age, frailty, and higher comorbidities [42,52]. Exclusion of patients who exhibit non-specific manifestations described earlier from surgery is of major concern [47] and defeats the main goal of preserving cognition, psychological functioning, and muscular strength, which in turn are important in maintaining quality of life and health in the elderly. With increased life expectancy today, persistent PHPT may chronically expose older patients to its metabolic effects [51]. Elderly patients referred for surgical intervention tend to have more severe symptoms, indicating that they are being presented for surgical intervention at a more advanced stage of the disease [2,52]. One ramification of a delay is a greater risk for the sequelae of PHPT, which includes increased risk of falls, fractures, kidney stones, renal failure, cardiovascular disease, and worsening neurocognitive symptoms, all of which have profound effect on the independence and wellbeing of an older person as well as on their family and caregivers [54,55,56]. Fractures are associated with considerable morbidity and mortality in this population. In addition, hip fractures contribute to long-term disability, functional impairment, poor quality of life, with these effects having greater implications in frail patients [2,24,57].

Current international guidelines recommend surgery in patients who have at least one of the following: (1) serum calcium > 1 mg/dL (0.25 mmol/L) above the upper limit of normal or (2) skeletal involvement: a fracture determined via vertebral fracture assessment or vertebral X-ray or (3) bone mineral density (BMD) via T-score ≤ −2.5 at any site or (4) renal involvement: eGFR or creatinine clearance < 60 mL/min or (5) nephrolithiasis or (6) hypercalciuria (>250 mg/day in women; >300 mg/day in men) or (7) age < 50 years [58]. While these guidelines focus on surgery in younger patients with PHPT, elderly asymptomatic patients are partially excluded from a parathyroidectomy. In addition, psychiatric symptoms are not considered an indication for surgery. Most recent studies have shown that surgery is safe and is the definitive treatment for PTPH in the elderly, even in those older than 80 years, with clinical outcomes similar to those of younger age groups [7,52]; members of the older age group do not have an increased risk of intra- and post-operative complications compared to younger patients [15,26,38,52,53,58,59].

Surgery is associated with improved BMD; decreased fragility fracture risk [35,45] (including patients without osteoporosis and with advanced frailty); general symptomatic relief [7]; and marked improvement in depressive mood, anxiety, psychomotor inhibition, and psychotic symptoms; and helps to preserve the quality of life [19,33]. Recent advancements in imaging and localization studies and the more widely available rapid intraoperative parathyroid hormone assay (IOPTH) (which assures that a focused parathyroidectomy has been adequately performed) have facilitated the adoption of minimally invasive approaches to parathyroidectomy, resulting in better outcomes [60,61,62].

On the other hand, data on pharmacotherapy (e.g., calcimimetics, bisphosphonates, antidepressants, antipsychotics) for older individuals with PHPT is scarce and often conflicting. Some data have shown that pharmacotherapy can result in normalization of calcium levels and improvement of BMD without an increase in the risk of fracture [34]. However, the adverse effects of the medications require special attention, particularly among frail older people who have an inherent risk of polypharmacy due to the need to treat multiple comorbidities, thus leading to various negative effects on their health due to the adverse actions from these drugs. In addition, the benefit of these medications in reducing calcium concentration only lasts while on therapy [47]. The benefit–risk ratio of surgery can be weighed in patients with limited life expectancy or high operative risk.

To the best of our knowledge, this is the only review on PHPT among the elderly population. However, we were able to include only 29 articles, most of which were observational. In addition, a selection bias might arise from restricting the research to the English language and to the most recent data (2013 to date), which may have limited the identification of non-English references or pertinent prior publications.

## 5. Conclusions

PHPT is a common yet overlooked and underdiagnosed condition among the older population, with symptoms that are often difficult to distinguish from the effects of aging itself. Delays and long intervals between the dates of diagnosis and treatment places the patient at elevated risk of complications, including osteoporosis, fractures, nephrolithiasis, and neuropsychiatric symptoms. PTX is the only cure for PHPT. The safety and efficacy of PTX in the older population in terms of improved BMD, fewer fractures, improved psychiatric symptoms, and improved quality of life is now well demonstrated in the literature. Advances in surgical techniques have brought minimal intra- and post-operative complications, with rates similar to those of the younger population. In the absence of evidence to suggest a significant effect from increased age and comorbidities on surgery complications and outcomes, the longstanding controversy around the need to operate for HPT in older patients is resolved. Prompt surgical intervention, a more effective and less costly solution, should always be offered unless there are contraindications.

## Figures and Tables

**Table 1:** Study characteristics on primary hyperparathyroidism in older population.

Study	Years	Study Design	Sample Size (N)	Mean Age (±SD) or Median Age Range (Years)
Seib et al. [40]	2023	Cohort	210206	N/A
Alobuia et al. [36]	2022	Cohort	210206	75.3 ± 6.8
Hangge et al. [44]	2022	Cross sectional	100 (<75 years) 100 (≥75 years)	63.3 (35.0–74.0) (<75 years) 78.8 (75.0–89.0) (≥75 years)
McCoy et al. [45]	2022	Cohort	187 (parathyroid exploration) 142 (total thyroidectomy)	63 ± 13 (parathyroid exploration) 54 ± 14 (total thyroidectomy)
Meng et al. [31]	2022	Case report	74	74
Papavramidis et al. [42]	2022	Cohort	96 (≤65 years) 38 (>65 years)	50.4 ± 9.8 (≤65 years) 72.1 ± 4.9 (>65 years)
Seib et al. [35]	2022	Cohort	210 206	75.0 ± 6.8
Herb et al. [47]	2021	Cross-sectional	94 803	76.0 (median)
Mueller et al. [26]	2021	Cohort	474 (<50) 1012 (50–64) 716 (65–74 years) 440 (≥75 years)	62.0 (median) (53–71)
O’sullivan et al. [37]	2021	Cross-sectional	202 (≥75 years) 1698 (<75 years)	59.7 (median)
Otsuki et al. [33]	2021	Case report	1	68.0
Seib et al. [41]	2021	Cohort	210,206	75.3 ± 6.8
Timmons et al. [29]	2021	Case report	87	87
Duskin-Bitan et al. [24]	2020	Cross-sectional	182	73 ± 4.0
Ekici et al. [49]	2020	Cross-sectional	64 (<65 years) 26 (>65 years)	52.2 ± 8.9 (<65 years) 71.4 ± 5.8 (>65 years)
Khokhar et al. [15]	2020	Cross-sectional	22220 (≤60 years) 22683 (61–79 years) 2798 (≥80 years)	49.7 ± 9.2 (≤60 years) 68.7 ± 5.1 (61–79 years) 82.9 ± 2.5(≥80 years)
Koman et al. [46]	2020	Cohort	19	77.0
Voci [30]	2020	Case report	76	76
Augusto et al. [28]	2019	Case report	75	75
Castellano et al. [27]	2019	Cross-sectional	212	72.3 ± 5.5
Lujan-Martinez et al. [32]	2019	Case report	74	74
Sluis et al. [34]	2019	Case series	8	88 ± 2.5
Dombrowsky et al. [23]	2018	Cross-sectional	25	84.0
Khan et al. [39]	2018	Case report	1	80.0
Seib et al. [48]	2018	Cross-sectional	13123 (≥40 years)	62.2 ± 11.0
Calo et al. [7]	2017	Cross-sectional	156 (<64 years) 76 (65–74 years) 24 (≥75 years)	51.5 ± 9.6 (<64 years) 69.2 ± 2.9 (65–74 years) 77.4 ± 3.4 (≥75 years)
Polistina et al. [43]	2017	Cross-sectional	135	73.0
Bajwa et al. [38]	2015	Case report	1	87.0
Denizot et al. [25]	2014	Cross-sectional	80 (<50 years) 89 (≥75 years)	N/A

Abbreviations: N/A: not available; SD: standard deviation.

**Table 2:** Main outcomes of primary hyperparathyroidism in the older population.

Study	Year	Outcomes
Seib et al. [40]	2023	Parathyroidectomy was associated with a lower long-term incidence of adverse CV outcomes when compared with nonoperative management.
Alobuia et al. [36]	2022	Racial/ethnic disparities exist in the management of PHPT among older adults.
Hangge et al. [44]	2022	BMD improves similarly in both cohorts with no difference in complication rates post parathyroidectomy.
McCoy et al. [45]	2022	Risk Analysis Index scores decreased after parathyroid exploration surgery, which reflects an improvement in frailty score.
Meng et al. [31]	2022	PHPT should be ruled out in a patient with new-onset psychosis.
Papavramidis et al. [42]	2022	Parathyroidectomy improves quality of life in both groups and frailty only in older group.
Seib et al. [35]	2022	Parathyroidectomy was associated with a lower risk of any fracture and hip fracture among older adults with PHPT.
Herb et al. [47]	2021	Parathyroidectomy is underused for symptomatic primary hyperparathyroidism in older adults.
Mueller et al. [26]	2021	Parathyroidectomy in elderly people have comparable risk of in-hospital complications compared with the younger population.
O’sullivan et al. [37]	2021	Elderly patients had lower calcium and PTH levels post-parathyroidectomy. In addition, surgery is considered safe for this population.
Otsuki et al. [33]	2021	PHPT can present with severe psychiatric symptoms, even in mild hypercalcemia. Those symptoms improve post-operatively.
Seib et al. [41]	2021	Older age, frailty, and multiple comorbidities were associated with nonoperative management in elderly patients with PHPT.
Timmons et al. [29]	2021	Mild hypercalcemia can influence cognitive function.
Duskin-Bitan et al. [24]	2020	Serum and urinary calcium decreased in patients aged 75 years and older who were treated conservatively.
Ekici et al. [49]	2020	Minimally invasive parathyroidectomy is safe in geriatric patients.
Khokhar et al. [15]	2020	Parathyroidectomy is a safe procedure in all age groups including people above 80 years old.
Koman et al. [46]	2020	Medical normalization of hypercalcemia can help in predicting outcome after parathyroidectomy.
Voci [30]	2020	Concomitant presence of cognitive dysfunction in an elderly patient can mask underlying PHPT.
Augusto et al. [28]	2019	Parkinsonism can have a significant remission after parathyroidectomy in patients with PHPT.
Castellano et al. [27]	2019	The clinical presentation of PHPT differs according to age, and this difference can influence the selection of management options.
Lujan-Martinez et al. [32]	2019	Cognitive impairment of the elderly secondary to hyperparathyroidism is overlooked.
Sluis et al. [34]	2019	Medical management is a reasonable option for PHPT patients over 85 years old.
Dombrowsky et al. [23]	2018	PHPT is underdiagnosed and undertreated in elderly patients.
Khan et al. [39]	2018	Primary hyperparathyroidism can rarely be secondary to a parathyroid cancer and adenoma synchronously.
Seib et al. [48]	2018	Frailty is associated with increased complications, reoperation, and prolonged LOS in patients undergoing parathyroidectomy for PHPT.
Calo et al. [7]	2017	Minimally invasive parathyroidectomy can be performed safely in elderly people.
Polistina et al. [43]	2017	Parathyroidectomy in elderly PHPT patients is safe, with a similar rate of morbidity to what is seen in younger population.
Bajwa et al. [38]	2015	There is limited research on the appropriate management of PHPT in very old individuals.
Denizot et al. [25]	2014	Parathyroidectomy is safe and curative for older adult with PHPT.

Abbreviations: PHPT: primary hyperparathyroidism; BMD: bone mineral density; CV: cardiovascular.

## Data Availability

The authors confirm that the data supporting the findings of this review is cited in both the text and the reference list.

## Appendix A

**Table A1:** JBI critical appraisal tool assessment for cross-sectional studies.

	Hangge et al., 2022 [44]	Herb et al., 2021 [47]	O’sullivan et al., 2021 [37]	Duskin-Bitan et al., 2020 [24]	Ekici et al., 2020 [49]	Khokhar et al., 2020 [15]	Castellano et al., 2019 [27]	Dombrowsky et al., 2018 [23]	Seib et al., 2018 [48]	Calo et al., 2017 [7]	Polistina 2017 [43]	Denizot et al., 2014 [25]
Were the criteria for inclusion in the sample clearly defined?	Yes	Yes	Yes	Yes	Not clear	Yes	Yes	Yes	Yes	Yes	Yes	Yes
Were the study subjects and the setting described in detail?	Yes	Yes	Yes	Yes	Yes	Yes	Yes	Yes	Yes	Yes	Yes	Not clear
Was the exposure measured in a valid and reliable way?	Yes	Yes	Yes	Yes	Yes	Yes	Yes	Yes	Yes	Yes	Yes	Yes
Were objective, standard criteria used for measurement of the condition?	Yes	Yes	Yes	Yes	Yes	Yes	Yes	Yes	Yes	Yes	Yes	Yes
Were confounding factors identified?	No	No	No	No	No	Yes	No	No	No	No	No	No
Were strategies to deal with confounding factors stated?	N/A	N/A	N/A	N/A	N/A	Yes	N/A	N/A	N/A	N/A	N/A	N/A
Were the outcomes measured in a valid and reliable way?	Yes	Yes	Yes	Yes	Yes	Yes	Yes	Yes	Yes	Yes	Yes	Yes
Was appropriate statistical analysis used?	Yes	Yes	Yes	Yes	Yes	Yes	Yes	Yes	Yes	Yes	Yes	Yes

Abbreviation: N/A: not applicable.

**Table A2:** JBI critical appraisal tool assessment for cohort studies.

	Seib et al., 2023 [40]	Alobuia 2022 [36]	Mccoy 2022 [45]	Papavramidis 2022 [42]	Seib et al., 2022 [35]	Mueller et al., 2021 [26]	Seib et al., 2021 [41]	Koman et al., 2020 [46]
Were the two groups similar and recruited from the same population?	Yes	Yes	No	Yes	Yes	Yes	Yes	Yes
Were the exposures measured similarly to assign people to both exposed and unexposed groups?	Yes	Yes	Yes	Yes	Yes	Yes	Yes	Yes
Was the exposure measured in a valid and reliable way?	Yes	Yes	Yes	Yes	Yes	Yes	Yes	Yes
Were confounding factors identified?	No	No	No	No	No	Yes	Yes	Yes
Were strategies to deal with confounding factors stated?	Yes	N/A	No	No	Yes	No	No	No
Were the groups/participants free of the outcome at the start of the study (or at the moment of exposure)?	Yes	Yes	Yes	Yes	Yes	Yes	Yes	Yes
Were the outcomes measured in a valid and reliable way?	Yes	Yes	Yes	Yes	Yes	Yes	Yes	Yes
Was the follow-up time reported and sufficient to be long enough for outcomes to occur?	Yes	No	Yes	Yes	Yes	Yes	Yes	Yes
Was follow-up complete, and if not, were the reasons for loss of follow-up described and explored?	Yes	No	Yes	No	Yes	Yes	Yes	Yes
Were strategies to address incomplete follow-up utilized?	No	No	No	No	No	N/A	No	No
Was appropriate statistical analysis used?	Yes	Yes	Yes	Yes	Yes	Yes	Yes	Yes

Abbreviation: N/A: not applicable.

**Table A3:** JBI critical appraisal tool assessment for case series.

	Sluis et al., 2019 [34]
Were there clear criteria for inclusion in the case series?	Yes
Was the condition measured in a standard, reliable way for all participants included in the case series?	Yes
Were valid methods used for identification of the condition for all participants included in the case series?	Yes
Did the case series have consecutive inclusion of participants?	No
Did the case series have complete inclusion of participants?	Yes
Was there clear reporting of the demographics of the participants in the study?	No
Was there clear reporting of clinical information of the participants?	Yes
Were the outcomes or follow-up results of cases clearly reported?	Yes
Was there clear reporting of the presenting site(s)/clinic(s) demographic information?	Yes
Was statistical analysis appropriate?	Yes

Abbreviation: N/A: not applicable.

**Table A4:** JBI critical appraisal tool assessment for case reports.

	Meng et al., 2022 [31]	Otsuki 2021 [33]	Timmons et al., 2021 [29]	Voci 2020 [30]	Augusto et al., 2019 [28]	Lujan et al., 2019 [32]	Khan et al., 2018 [39]	Bajwa et al., 2015 [38]
Were patient’s demographic characteristics clearly described?	Yes	Yes	Yes	Yes	No	No	Yes	Yes
Was the patient’s history clearly described and presented as a timeline?	Yes	Yes	Yes	Yes	Yes	Yes	Yes	Yes
Was the current clinical condition of the patient on presentation clearly described?	Yes	Yes	Yes	Yes	Yes	Yes	Yes	Yes
Were diagnostic tests or assessment methods and the results clearly described?	Yes	Yes	Yes	Yes	Yes	Yes	Yes	Yes
Was the intervention(s) or treatment procedure(s) clearly described?	No	Yes	Yes	Yes	Yes	No	Yes	Yes
Was the post-intervention clinical condition clearly described?	Yes	Yes	Yes	Yes	Yes	Yes	Yes	Yes
Were adverse events (harms) or unanticipated events identified and described?	No	No	No	No	No	No	No	Yes
Does the case report provide takeaway lessons?	Yes	Yes	Yes	Yes	Yes	Yes	Yes	Yes

Abbreviation: N/A: not applicable.
